# Removal of Hg^0^ from simulated flue gas over silver-loaded rice husk gasification char

**DOI:** 10.1098/rsos.180248

**Published:** 2018-09-12

**Authors:** Ru Yang, Yongfa Diao, Befkadu Abayneh

**Affiliations:** 1 College of Environmental Science and Engineering, Donghua University, Shanghai 201620, People's Republic of China; 2 College of Energy and Architectural engineering, Henan University of Urba Construction, Pingdingshan 467001, People's Republic of China

**Keywords:** Hg^0^, silver-loaded rice husk gasification char, adsorption performance, adsorption–regeneration

## Abstract

Mercury released into the atmosphere from coal combustion is harmful to humans and the environment. Rice husk gasification char (RHGC) is an industrial waste of biomass gasification power generation, which is silver-loaded to develop a novel and efficient sorbent for mercury removal from simulated flue gas. The experiment was carried out in a fixed-bed experimental system. The Hg^0^ adsorption performance of RHGC was improved significantly after loading silver. Hg^0^ adsorption capacity and mercury inlet concentration were found to be nonlinear. The adsorption capacity of RHGC decreased with the increase of reaction temperature. SO_2_ inhibited mercury removal, NO and HCl promoted mercury removal; the Hg^0^ adsorption capacity in the simulated flue gas was higher than that in pure N_2_. The silver-loaded rice husk gasification char (SRHGC) could be recycled about five times without significantly losing its removal efficiency. The SRHGC will not only reduce the cost of mercury removal but also save energy and reduce environmental pollution. At the same time, it provides a new way for the resource utilization of RHGC.

## 1. Introduction

With the rapid development of the global economy, the demand for coal is increasing rapidly. China has been the largest coal-consuming country in the world consuming more than 3 billion tons in 2010. Coal combustion produces serious environmental and ecological damage as it releases SO_2_, NO_x_, mercury and other pollutants to the atmosphere. Therefore, controlling pollutant emissions from a coal-fired plant has received wider attention all over the world [1]. Even though at present the emission control technology for SO_2_ and NO_x_ is relatively mature, the trace elemental mercury emission control research and applications are still at starting stage. Elemental mercury has high toxicity, volatility, persistence and bioaccumulation in the environment [2,3], it will also attack the neurons in the human nervous system causing damage to the brain, spinal cord, and nerves once it enters the human body [4,5]. Therefore, controlling mercury emission has become a global hot issue [2,6].

Mercury has three forms during combustion: particle-bound atoms (Hg^p^), oxidized forms (Hg^2+^) and elemental mercury Hg^0^ vapour in coal-fired flue gas [7]. Hg^p^ can be removed by particulate matter (PM) controlling devices such as electrostatic precipitators and bag filter. Hg^2+^ is relatively stable and can be removed by wet flue gas desulfurization device (WFGD). Hg^0^ is volatile and insoluble in water, so it is not easy to separate it with traditional methods [8]. On the contrary, the concentration of Hg^0^ is rising in WFGD, because sulfite reduces some of the Hg^2+^ in the washing solution. Therefore, finding a mechanism for capturing of elemental mercury is the key to controlling mercury pollution [9].

Different adsorbents, such as activated carbon (AC) [10–13], calcium-based sorbents [14,15], noble metals and transition metals [16–19], metal oxides [20], fly ash [21] and zeolite [22], have been tested for Hg^0^ removal. Among them, AC injection is widely used in coal-fired plant mercury emission control. However, the high cost and its negative effect on the usage of fly ash limited its application in power plants [23,24]. Therefore, there is a need for developing cost-effective, more efficient and renewable sorbents to replace the expensive materials in industrial application.

Biomass gasification power generation has the characteristics of environmental protection and regeneration, especially the current high oil prices, which all attracted the attention of many governments. As a clean and renewable resource, the harmful biomass substance (sulfur, ash and so forth) content of rice husk is only one-tenth of coal. Comparing coal with biomass, the net CO_2_ emission can be reduced by about 84–93% per unit heating value when they burn [25]. Biomass gasification power generation is a potential alternative for reducing greenhouse gas emissions and preventing global environmental degradation. The global biomass gasification power generation installed capacity had reached 39 GW, and the annual generating capacity reached about 200 billion kW h in 2004 [26]. The installed capacity of biomass gasification power generation has reached 10 GW in America; China will reach 30 GW in 2020 [27,28]. Biomass gasification and power generation technology has brought great prospects for straw utilization and storage.

Biomass gasification char is an industrial waste of biomass gasification power generation; it is significantly cheaper than a manufactured sorbent. Char has a loose physical structure and highly active functional groups on the surface [29,30]. The high carbon content and well-developed micro-porosity of biomass gasification chars together with their high chlorine and aluminium content play a role in improving their mercury retention capacity [31].

As biomass gasification char has a strong loading capacity, mechanical strength and recycling capacity, it has a real potential to replace AC as a sorbent for mercury removal. The brominated biomass ash was characterized and tested for mercury capture, and release at high temperatures; the tests of the new brominated sorbent in a 375 MW coal-fired power plant showed promising performance [32]. After the rice husk char (RHC) had been modified with ammonium halides, the mercury removal performance was significantly enhanced [33]. Using the sol–gel method to prepare elm char/TiO_2_ photocatalytic materials, the elm char/TiO_2_ had a high removal efficiency to mercury under UV, the oxygen and water vapour were positive to mercury removal, the mercury removal efficiency can reach 86% with 10% oxygen in the atmosphere under UV light [34]. Rice husk was pyrolysed, activated by CO_2_ and H_3_PO_4_ and modified by NH_4_Br separately to prepare RHC, rice husk-activated char and rice husk-modified char as sorbents for removal of mercury. After modification, the Hg^0^ removal efficiency of the rice husk-pyrolysed char is improved over 60%, while the activated char is increased by 90%; the mercury adsorption efficiency of CO_2_ and H_3_PO_4_-activated cokes remains above 90% and 80%, respectively, after 120 min of adsorption [35].

Silver reacts with mercury vapour to produce silver amalgam; silver amalgam can be decomposed and release mercury at a certain temperature so that the adsorbent can be used again. At present, nano-silver particles are loaded on the surface or inside of the adsorbent, such as silver-loaded AC [36,37] and silver-loaded zeolite [19]; they are not used in large-scale application to remove mercury because of the high price. At present, there have been no reports that use silver-loaded biomass gasification char for mercury removal in the coal-fired flue gas. Using an industrial waste from the biomass gasification power plant and loading silver on it to remove mercury is potential. Using silver-loaded rice husk gasification char (SRHGC) for mercury removal and regenerating it after heating, will not only reduce the cost of mercury removal but also save energy and reduce environmental pollution. At the same time, it provides a new way for the resource utilization of rice husk gasification char (RHGC).

## 2. Material and methods

### 2.1. Adsorbent preparation

RHGC was collected from Jiangsu Gaoyou Straw Gasification Power Plant, China. In order to determine whether RHGC can be used as Hg^0^ removal adsorbent by measuring the elemental content and pore structure parameters of RHGC, the elemental content was analysed by the elemental analyser (Vario EL cube, Elmentar, Germany), the result was as follows: C 43.58%, H 1.84%, S 0.30%, N 1.59%, O 21.82%, K 6.52%, Si 3.92%, Ca 1.36%, Cl 0.90%, P 0.29%, Al 0.14%. C is an excellent adsorbent for Hg^0^. The high carbon content of SRHGC provides an important basis for the removal of Hg^0^. The O element content is 21.82%, which is second only to the element C, a large number of O and C elements will form C–O and C=O oxygen-containing functional groups, which have strong oxidative properties and will play a key role in promoting Hg^0^ removal. The pore structure parameters of RHGC were measured by the automatic surface and pore analyser (ASAP2020, Microneter, America), the pore surface area was 60.36 m^2^ g^−1^, the aperture was 6.34 nm, the pore volume was 0.042 m^3^ g^−1^, which belonged to mesoporous materials. Mesoporous is the molecular channel of the adsorbed material. Under the certain relative pressure, the gas is adsorbed into the pore through the mechanism of agglomeration to achieve a good adsorption effect. So, RHGC could be used as adsorbent for removing mercury.

RHGC was dried, ground and sieved to retain the particles within 80–120 mesh. Impregnation of the selected RHGC with hydrochloric acid was achieved by treating the RHGC with hydrochloric acid (purity: 20%) for 30 min, then vacuum-dried for 2 h at 50°C and cooled down to room temperature, washed with deionized water to neutrality and then dried. Six millilitre AgNO_3_ solution with a mass concentration of 2 mg ml^−1^ (purity: 97%) was taken to place in the beaker, 300 mg RHGC was added and the pH was adjusted to 9.7 with ammonia (purity: 97%), oscillated for 24 h under 298 K constant temperature. The RHGC was filtered and placed in the electric furnace tube and heated for 4 h at 120°C in a nitrogen atmosphere, the samples were then taken out after cooling them down to room temperature, and are now a SRHGC. Using 300 mg SRHGC as adsorbent, adsorption temperature was about 160°C, flue gas flow rate was 0.003 m^3^ min^−1^, mercury inlet concentration was about 38.6 μg m^−3^ and the adsorption time was 120 min. X-ray photoelectron spectroscopy (XPS) and scanning electron microscopy and energy-dispersive spectrometry (SEM-EDS) were used to characterize RHGC.

### 2.2. Experimental set-up

**Figure 1.**
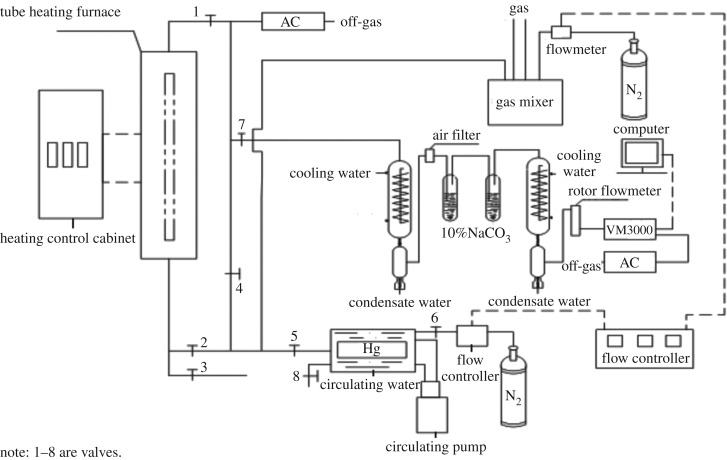
Schematic diagram of fixed-bed device.

The experiment was conducted in a fixed-bed experimental set-up; it consisted of fixed-bed reaction system, Hg^0^ vapour production system, flow controlling system, flue gas heating system, off-gas treatment system, and so forth, as shown in figure 1.

A constant flow rate of various gas components was controlled by a flow meter. The temperature was provided by a tube heating furnace which was controlled by a heating control cabinet (accuracy was ±1°C). The reaction outlet gas was detected online by mercury concentration detector (VM3000 mercury concentration detector, accuracy 0.1 μg m^−3^, Mercury Instruments Co., Germany). The Hg^0^ production system produced the desired Hg^0^ vapour concentration under constant circulating water (35°C). Nitrogen gas (N_2_) was used as the carrier gas to carry the Hg^0^ vapour into the experimental system. An adsorption tank equipped with AC was used as the off-gas treatment section for adsorbing mercury which was not adsorbed. While using 300 mg SRHGC as adsorbents, adsorption temperature was about 160°C, flue gas flow rate was 0.003 m^3^ min^−1^, mercury inlet concentration was about 38.6 μg m^−3^, the adsorption time was 120 min.

The mercury removal efficiency could be calculated by the comparison between inlet and outlet total mercury concentration by the following equation:

2.1 $$\tau =\frac{{\left[\text{H}{\text{g}}^{0}\right]}_{\mathrm{out}}}{{\left[\text{H}{\text{g}}^{0}\right]}_{\mathrm{in}}},$$

and

2.2 $$Q=\frac{1}{m}\int_{t_{1}}^{t_{2}}\left(1-\frac{C}{C_{0}}\right)qC_{0}d_{t}\approx \sum_{i=0}^{n}\left(1-\frac{C_{i}+C_{i+1}}{2C_{0}}\right)q\Delta t\cdot \frac{C_{0}}{m},$$

where *τ* is the mercury breakthrough rate (%), [Hg^0^]_in_ and [Hg^0^]_out_ stand for the inlet and outlet concentrations of mercury (μg m^−3^), respectively, *Q* represents the mercury adsorption capacity (μg g^−1^) in 120 min, *C*_0_ is the inlet concentration of mercury, *q* is the gas flow rate (m^3^ min^−1^), *m* is the mass of adsorbents (g), Δ*t* is the time interval and Δ*t* = 10.

Before each test, blank test without adsorbent was first conducted to examine any possible interaction between the flue gas components and Hg^0^.

## 3. Results and discussion

### 3.1. Characterization of sorbents

**Figure 2.**
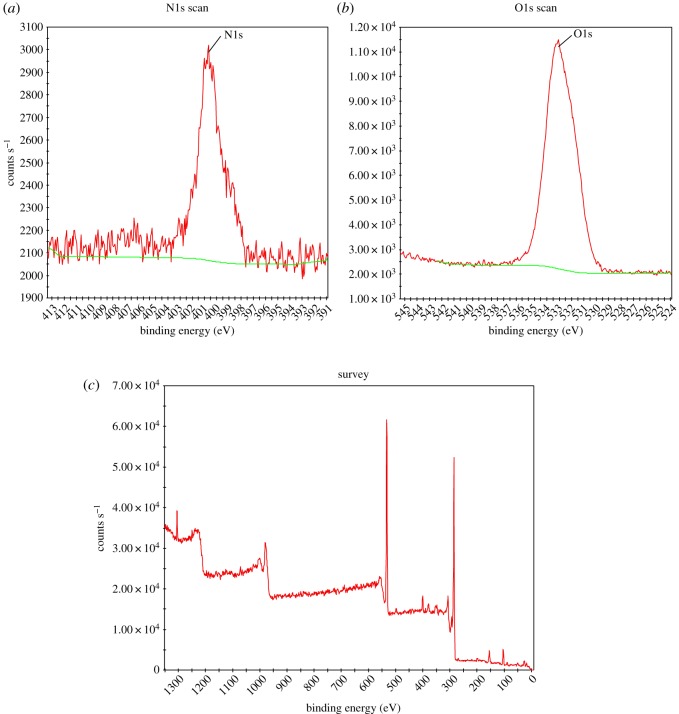
(*a*) The fitting curves of N1s, (*b*) the fitting curves of O1s and (*c*) XPS of RHGC.

RHGC was characterized by XPS (Escalab 250X, Thermo Scientific, America), the result is shown in figure 2*a*,*b* were the fitting curves of O1s and N1s, respectively. It could be seen that the peak at 533.1 eV corresponded to the oxygen bounded to the carbon single bond in ether oxygen, ester and anhydride structures [38,39]; the peak position of N1s at 400.6 eV corresponded to the nitrogen peak of pyrrole; the pyrrole functional group could promote mercury removal through catalysis [40]. The oxygen-containing functional groups and nitrogen-containing functional groups in the adsorbent play an important role in mercury removal process.

**Figure 3.**
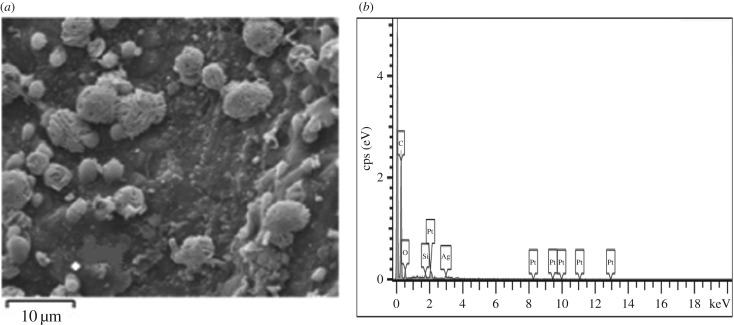
SEM-EDS of SRHGC. (*a*) Spatial distributions of SRHGC, (*b*) element type and content of SRHGC.

SRHGC was analysed by SEM-EDS to judge element type, content and spatial distributions in the samples. The characteristic peaks of C, Si, O, Ag and Pt confirmed that the SRHGC samples were composed of C, Si, O, Ag and Pt elements, respectively, as can be seen from figure 3. The pore structure was relatively developed, which provided favourable conditions for elemental mercury removal.

### 3.2. The Hg^0^ removal efficiency of adsorbents

**Figure 4.**
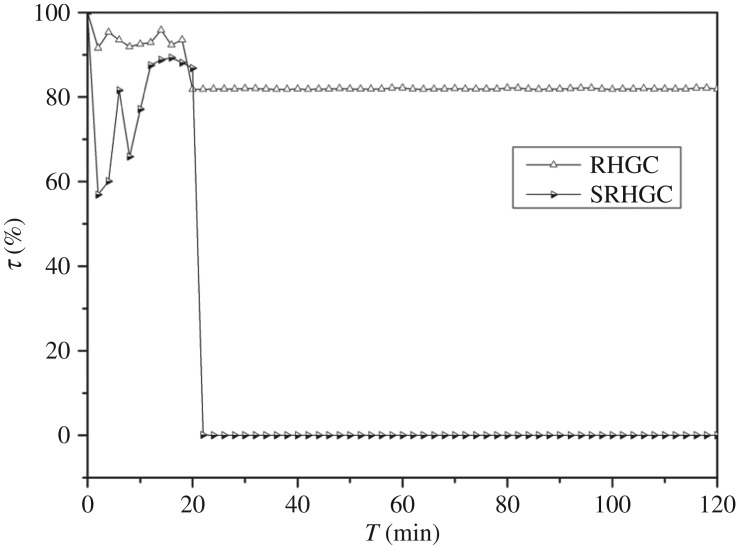
Breakthrough curve of mercury adsorption of RHGC and SRHGC in pure N_2._ The experimental conditions: adsorption temperature was about 160°C, flue gas flow rate was 0.003 m^3^ min^−1^, mercury inlet concentration was about 38.6 μg m^−3^, the adsorption time was 120 min.

The breakthrough curve of mercury adsorption of RHGC (300 mg) and SRHGC (300 mg) in pure N_2_ are shown in figure 4. After 20 min, the breakthrough rate of mercury adsorption was basically stable, the breakthrough rate of RHGC was 81.8%, SRHGC was basically penetrated, so the Hg^0^ adsorption performance of RHGC was improved significantly after loading silver. The silver in the SRHGC had a strong affinity to mercury and could generate silver amalgam, thereby improving the Hg^0^ removal efficiency. As a low-cost industrial waste, SRGHC becomes a potential adsorbent for mercury removal.

### 3.3. Effect of mercury inlet concentration on Hg^0^ adsorption capacity of silver-loaded rice husk gasification char

**Figure 5.**
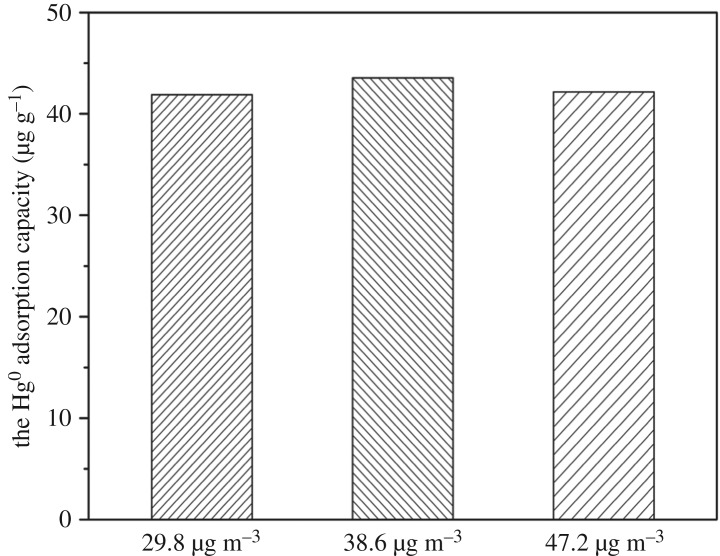
Effect of mercury inlet concentration on Hg^0^ adsorption capacity of SRHGC. The experimental conditions: adsorption temperature was about 160°C, flue gas flow rate was 0.003 m^3^ min^−1^, the adsorption time was 120 min.

The effect of mercury inlet concentration on the Hg^0^ adsorption capacity of SRHGC is shown in figure 5. When the [Hg^0^]_in_ increased from 29.8 to 38.6 µg m^−3^, the Hg^0^ adsorption capacity rose from 41.87 to 43.54 µg g^−1^. However, when the [Hg^0^]_in_ rose from 38.6 to 47.2 µg m^−3^, the Hg^0^ removal capacity decreased from 43.54 to 42.16 µg g^−1^. In the three mercury inlet concentrations of this experiment, the Hg^0^ adsorption capacity and the [Hg^0^]_in_ did not show a linear relationship. There might be two reasons for this: on the one hand, an increase of mercury inlet concentration could have led to improved mercury removal ability by increasing chances of mercury adsorption, which is a positive removal effect. On the other hand, with the increase of the mercury inlet concentration, more active sites, and adsorption vacancies will be required, which resulted in a reduced adsorption capacity, which is a negative efficiency. When the positive efficiency is more than the negative efficiency, the adsorption capacity increased; on the contrary, the adsorption capacity will decrease.

### 3.4. Effect of adsorption temperature on the Hg^0^ adsorption capacity of silver-loaded rice husk gasification char

**Figure 6.**
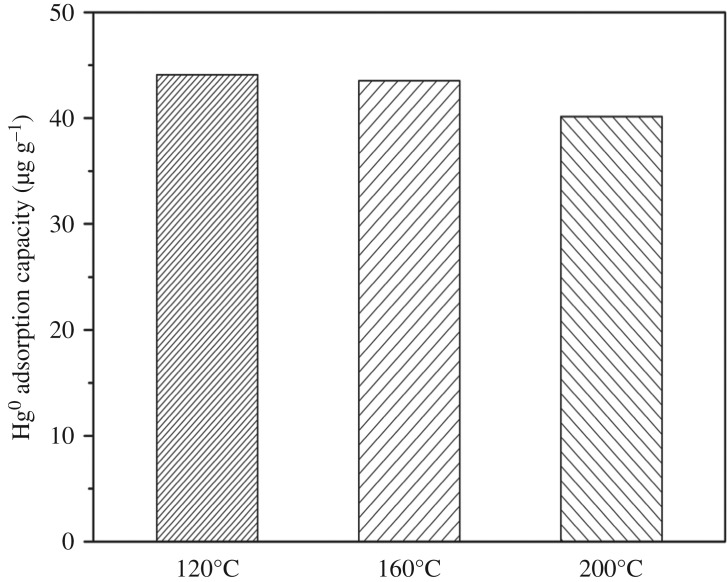
Effect of adsorption temperature on the Hg^0^ adsorption capacity of SRHGC. The experimental conditions: flue gas flow rate was 0.003 m^3^ min^−1^, mercury inlet concentration was about 38.6 µg m^−3^, the adsorption time was 120 min.

Adsorption capacity under different flue gas temperature is shown in figure 6. When the reaction temperature increased from 120 to 200°C, the Hg^0^ adsorption capacity of SRHGC decreased from 44.09 to 41.15 μg g^−1^. Physical and chemical adsorption of mercury occurs on the surface of SRHGC. Under lower temperature conditions, physical adsorption plays a more vital role than chemical adsorption, as there are many vacant adsorption spaces on the surface of SRHGC to which gaseous mercury would give priority. On the contrary, when temperature increases, the chemical adsorption rate will be higher than physical adsorption rate as the activation energies will be increased. The chemical bonds of the oxygen-containing functional groups might be broken, and the surface of the SRHGC might be degraded because of high temperature, which resulted in a decrease in chemical adsorption. Therefore, the adsorption capacity of SRHGC reduced. The higher the temperature, the faster will be the decline in adsorption.

### 3.5. Effect of different gas compositions on the Hg^0^ adsorption capacity of silver-loaded rice husk gasification char

**Figure 7.**
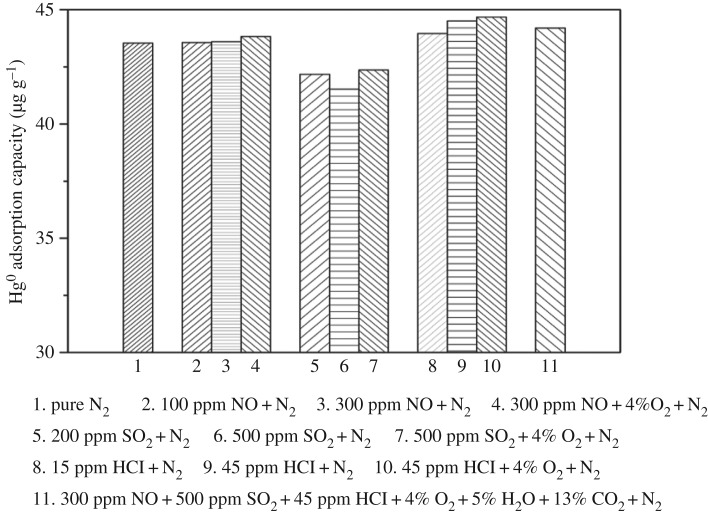
Effect of different gas compositions on the Hg^0^ adsorption capacity of SRHGC. The experimental conditions: adsorption temperature was about 160°C, flue gas flow rate was 0.003 m^3^ min^−1^, mercury inlet concentration was about 38.6 µg m^−3^, the adsorption time was 120 min.

The effect of various gas compositions on the Hg^0^ adsorption capacity of SRHGC was investigated, including O_2_, NO, SO_2_, HCl and simulated flue gas, the result is shown in figure 7.

#### 3.5.1. Effect of NO on Hg^0^ adsorption capacity

As can be seen from figure 7, the Hg^0^ adsorption capacity under pure N_2_ was around 43.54 μg g^−1^. When 100 ppm NO was added to the N_2_ gas stream, the Hg^0^ adsorption capacity increased from 43.54 to 43.57 μg g^−1^, and further increased when NO concentration was increased to 300 ppm. Moreover, the adsorption capacity was 43.83 μg g^−1^ in the presence of 4% O_2_. NO has lower affinity to adsorbent [41,42], but when NO and O_2_ coexisted, a strong oxidant NO_2_ was formed. The following reaction equations could explain this:

3.1 $$\text{H}{\text{g}}^{0}+\text{absorbent}\;\text{surface}\to \text{H}{\text{g}}^{0}(\text{ad}),$$

3.2 $$\text{H}{\text{g}}^{0}+\text{surface}-\text{O}\to \text{surface}-\text{O}-\text{Hg, }$$

3.3 $$\text{NO}+\frac{1}{2}{\text{O}}_{2}\to \text{N}{\text{O}}_{2},$$

3.4 $$\text{N}{\text{O}}_{2}+\text{H}{\text{g}}^{0}\to \text{HgO}+\text{NO}$$

3.5 $$\;\text{and}\;\text{HgO}+\text{NO}+\frac{3}{2}{\text{O}}_{2}\to \text{Hg }{\left(\text{N}{\text{O}}_{3}\right)}_{2}.$$

#### 3.5.2. Effect of SO_2_ on the Hg^0^ adsorption capacity

As illustrated in figure 7, the Hg^0^ adsorption capacity decreased from 43.54 to 42.18 μg g^−1^ when 200 ppm SO_2_ was added to the pure N_2_; the Hg^0^ adsorption capacity further reduced when SO_2_ was increased to 500 ppm. The Hg^0^ adsorption capacity was about 42.36 μg g^−1^ when 4% O_2_ was added. SO_2_ might compete with Hg^0^ for the active site on the surface of SRHGC, which inhibited the Hg^0^ adsorption and the Ag-amalgam formation. Therefore, the addition of SO_2_ lowered Hg^0^ adsorption capacity.

#### 3.5.3. Effect of HCl on the Hg^0^ adsorption capacity

When 15 ppm HCl was added to the pure N_2_ gas stream, the Hg^0^ adsorption capacity increased from 43.54 to 43.95 μg g^−1^ and further raised to 44.51 μg g^−1^. When 45 ppm HCl was added, Hg^0^ adsorption capacity was around 44.68 μg g^−1^ in the presence of 4% O_2_. Therefore, HCl could bring a significant enhancement to Hg^0^ adsorption capacity. There were two possible mechanisms, on the one hand, when HCl entered the SRHGC, a part of HCl may participate in a chemical reaction, which will increase active areas, and consequently, Hg^0^ adsorption capacity increases. On the other hand, the reaction of HCl and Hg^0^ will form HgCl_2_, which promoted Hg^0^ adsorption capacity. When HCl gas entered into the adsorbent layer, HCl and Hg^0^ would react to form HgCl and finally would form a stable HgCl_2_, thereby enhancing the adsorption capacity of the adsorbent. The adsorption mechanism could be described by equations (3.6–3.10):

3.6 $$2\text{H}{\text{g}}^{0}(\text{g})+4\text{HCl }(\text{g})+{\text{O}}_{2}(\text{g})\to 2\text{HgC}{\text{l}}_{2}(\text{g},\text{\textbackslash{};s})+2{\text{H}}_{2}\text{O }(\text{g}),$$

3.7 HCl (g)→Cl(g)+H, 

3.8 $$\text{H}{\text{g}}^{0}(\text{g})+2\text{Cl}(\text{g})\to \text{HgC}{\text{l}}_{2}(\text{s},\text{g}),$$

3.9 $$2\text{Cl}(\text{g})\to \text{C}{\text{l}}_{2}(\text{g})$$

3.10 $$\;\text{and}\;\text{H}{\text{g}}^{0}(\text{g})+\text{C}{\text{l}}_{2}(\text{g})\to \text{HgC}{\text{l}}_{2}(\text{g, s}).$$

#### 3.5.4. Hg^0^ adsorption performance of SRHGC in simulated flue gas

The simulated flue gas was composed of 300 ppm NO, 500 ppm SO_2_, 45 ppm HCl, 4% O_2_, 5% H_2_O, 13% CO_2_ and N_2_. In simulated flue gas, H_2_O would compete with Hg^0^ for the same adsorption site on the surface of adsorbent [43,44]; on the other hand, H_2_O would promote Hg^0^ adsorption when SO_2_ and O_2_ coexisted, the possible chemical reactions are shown in the following reaction equations:

3.11 $${\text{H}}_{2}\text{O}(\text{g})+\text{H}{\text{g}}^{0}(\text{ads})\to {\text{H}}_{2}\text{O}(\text{ads})+\text{H}{\text{g}}^{0}(\text{g}),$$

3.12 $$\text{S}{\text{O}}_{2}+\frac{1}{2}{\text{O}}_{2}\to \text{S}{\text{O}}_{3},$$

3.13 $$\text{S}{\text{O}}_{3}+{\text{H}}_{2}\text{O}\to {\text{H}}_{2}\text{S}{\text{O}}_{4\;},$$

3.14 $$\text{H}{\text{g}}^{0}+\frac{1}{2}{\text{O}}_{2}\to \text{HgO}$$

3.15 $$\;\text{and}\;\text{HgO }+{\text{H}}_{2}\text{S}{\text{O}}_{4}\to \text{HgS}{\text{O}}_{4}+{\text{H}}_{2}\text{O}.$$

As can be seen from figure 7 (the 11th column chart), Hg^0^ adsorption capacity was 44.2 μg g^−1^ in simulated flue gas; it was higher than Hg^0^ adsorption capacity of pure N_2_. Hg^0^ adsorption performance of SRHGC was not a simple superposition of a single gas in simulated flue gas, but a result of the synergistic effect of various flue gas and SRHGC.

### 3.6. Adsorption–regeneration cycles of SRHGC

The adsorption–regeneration cycles of SRHGC were investigated. SRHGC was 300 mg, the adsorption temperature was about 160°C, flue gas flow rate was 0.003 m^3^ min^−1^, mercury inlet concentration was about 38.6 μg m^−3^, the adsorption time was 120 min.

**Figure 8.**
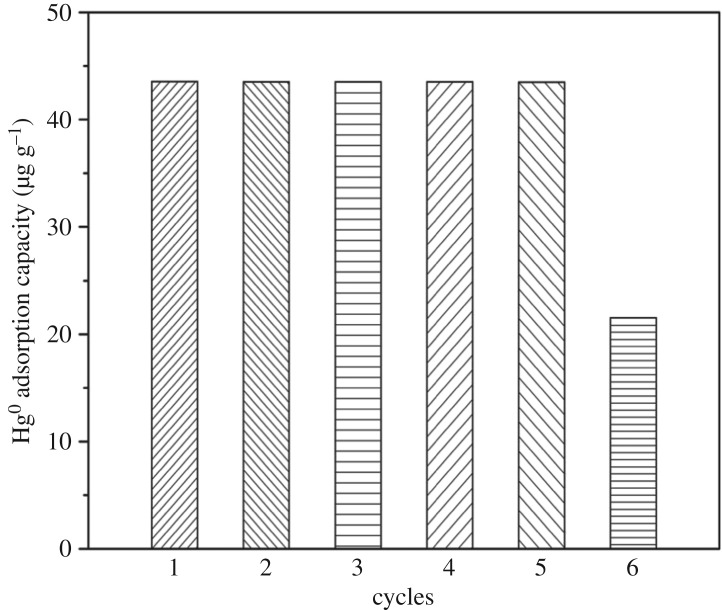
Adsorption–regeneration cycles of SRHGC. The experimental conditions: adsorption temperature was about 160°C regeneration temperature was about 350°C, flue gas flow rate was 0.003 m^3^ min^−1^, mercury inlet concentration was about 38.6 µg m^−3^, the adsorption time was 120 min.

The regeneration temperature was 350°C. After adsorption for 2 h, it switched to the regeneration pipeline, when the mercury analyser showed that the mercury concentration was below 3.86 µg m^−3^, switched back to the adsorption pipeline, circulated the operation, calculated Hg^0^ adsorption capacity, the number of regeneration was judged by Hg^0^ adsorption capacity, the adsorbent was cooled to room temperature for reuse after regeneration, the results are shown in figure 8.

Figure 8 indicates that the Hg^0^ adsorption capacity of the SRHGC decreased significantly at the sixth adsorption–regeneration cycle compared to the first five adsorption–regeneration cycles. Therefore, the SRHGC could be recycled for about five times. The silver and mercury could generate silver amalgam; the silver amalgam was a physical combination which could easily be separated from each other. Mercury vapour separated and discharged from the SRHGC so that the adsorbent could regenerate and recover its adsorption performance. But increasing the number of cycles will reduce the regeneration capacity of SRHGC.

## 4. Conclusion

A novel method of SRHGC for Hg^0^ removal in the simulated flue gas was developed. The SRHGC could be regenerated by heating, which would not only reduce the cost but also save energy and reduce environmental pollution. At the same time, it also created a new use or function for the RHGC. The experiment was conducted in a fixed-bed experimental system. The following conclusions were made:

(1) The Hg^0^ removal performance was significantly improved after the RHGC was loaded with silver.
(2) The Hg^0^ adsorption capacity and the mercury inlet concentration did not have a linear relationship, with an increase in initial concentration the adsorption increased first and then decreased.
(3) In the experimental range, the adsorption capacity of SRHGC decreased with the increase of reaction temperature.
(4) SO_2_ inhibited mercury removal; however, NO and HCl promoted mercury removal; the Hg^0^ adsorption performance was a result of synergistic effect in simulated flue gas.
The SRHGC could be recycled about five times.

## Supplementary Material

Electronic supplementary material

## Supplementary Material

Electronic supplementary material

## Data accessibility

The datasets supporting this article have been uploaded as part of the electronic supplementary material.
