# Supplementary material for: Removal of Hg0 from simulated flue gas over silver-loaded rice husk gasification char
Source: R Soc Open Sci. 2018 Sep 12;5(9):180248. doi: 10.1098/rsos.180248 (PMC6170578; doi:10.1098/rsos.180248)
Supplement: Electronic supplementary material [file rsos180248supp1.doc]

Removal of Hg0 from simulated flue gas over sliver-loaded rice husk gasification char

**Ru Yang^1,2^, Yongfa Diao^1^, Befkadu Abayneh^1^**

1. *College of Environmental Science and Engineering, Donghua University, Shanghai 201620, China*
2. *College of Energy and Architectural engineering, Henan University of Urba Construction, Pingdingshan, 467001, China.*

**1. Breakthrough curve of mercury adsorption of RHGC and SRHGC in pure N_2_**

| T(min) | RHGC  τ(%) | SRHGC  τ(%) |
| --- | --- | --- |
| 0 | 100 | 0 |
| 2 | 91.6 | 12.3 |
| 4 | 95.3 | 14.7 |
| 6 | 93.5 | 18.9 |
| 8 | 91.9 | 13.5 |
| 10 | 92.5 | 14.1 |
| 12 | 92.9 | 12.5 |
| 14 | 95.8 | 11.2 |
| 16 | 92.3 | 10.7 |
| 18 | 93.5 | 11.9 |
| 20 | 81.8 | 13.2 |
| 22 | 81.8 | 0.1 |
| 24 | 81.9 | 0 |
| 26 | 81.9 | 0 |
| 28 | 81.9 | 0 |
| 30 | 82 | 0 |
| 32 | 82 | 0 |
| 34 | 81.9 | 0 |
| 36 | 81.8 | 0 |
| 38 | 81.8 | 0 |
| 40 | 81.9 | 0 |
| 42 | 81.8 | 0 |
| 44 | 81.9 | 0 |
| 46 | 81.9 | 0 |
| 48 | 82 | 0 |
| 50 | 81.9 | 0 |
| 52 | 81.9 | 0 |
| 54 | 81.9 | 0 |
| 56 | 81.9 | 0 |
| 58 | 82.1 | 0 |
| 60 | 82.1 | 0 |
| 62 | 81.9 | 0 |
| 64 | 81.8 | 0 |
| 66 | 81.9 | 0 |
| 68 | 81.9 | 0 |
| 70 | 82 | 0 |
| 72 | 81.9 | 0 |
| 74 | 81.9 | 0 |
| 76 | 81.9 | 0 |
| 78 | 81.9 | 0 |
| 80 | 82.1 | 0 |
| 82 | 82.1 | 0 |
| 84 | 81.9 | 0 |
| 86 | 81.8 | 0 |
| 88 | 81.9 | 0 |
| 90 | 81.9 | 0 |
| 92 | 82 | 0 |
| 94 | 82.1 | 0 |
| 96 | 82.1 | 0 |
| 98 | 81.9 | 0 |
| 100 | 81.8 | 0 |
| 102 | 81.9 | 0 |
| 104 | 81.9 | 0 |
| 106 | 82 | 0 |
| 108 | 81.9 | 0 |
| 110 | 81.9 | 0 |
| 112 | 81.9 | 0 |
| 114 | 81.9 | 0 |
| 116 | 82.1 | 0 |
| 118 | 82.1 | 0 |
| 120 | 81.9 | 0 |

**2.Effect of mercury inlet concentration on Hg^0^ adsorption capacity of SRHGC**

| Mercury inlet concentration(μg/m^3^) | 29.8 | 38.6 | 47.2 |
| --- | --- | --- | --- |
| Hg^0^ adsorption capacity(μg/g) | 41.87 | 43.54 | 42.16 |

**3.Effect of adsorption temperature on the Hg^0^ adsorption capacity of SRHGC**

| Different flue gas temperature(℃) | 120℃ | 160℃ | 200℃ |
| --- | --- | --- | --- |
| Hg^0^ adsorption capacity(μg/g) | 44.09 | 43.54 | 41.15 |

**4.Effect of different gas compositions on the Hg^0^ adsorption capacity of SRHGC**

| Different flue gas compositions | Hg^0^ adsorption capacity(μg/g) |
| --- | --- |
| pureN_2_ | 43.54 |
| 100ppmNO+N_2_ | 43.57 |
| 300ppmNO+N_2_ | 43.61 |
| 300ppmNO+4%O_2_+N_2_ | 43.83 |
| 200ppmSO_2_+N_2_ | 42.18 |
| 500ppmSO_2_+N_2_ | 41.52 |
| 500ppmSO_2_+4%O_2_+N_2_ | 42.36 |
| 15ppmHCL+N_2_ | 43.95 |
| 45ppmHCL+N_2_ | 44.51 |
| 45ppmHCL+4%O_2_+N_2_ | 44.68 |
| 300ppmNO+500ppmSO_2_+45ppmHCL+4%O_2_+5%H_2_O+13%CO_2_+N_2_ | 44.2 |

**5.Adsorption-regeneration cycles of SRHGC**

| Adsorption-regeneration cycles | 1 | 2 | 3 | 4 | 5 | 6 |
| --- | --- | --- | --- | --- | --- | --- |
| Hg^0^ adsorption capacity (μg/g) | 43.54 | 43.54 | 43.54 | 43.54 | 43.54 | 22.1 |
