# Supplementary figures and images for: Removal of Hg0 from simulated flue gas over silver-loaded rice husk gasification char
Source: R Soc Open Sci. 2018 Sep 12;5(9):180248. doi: 10.1098/rsos.180248 (PMC6170578; doi:10.1098/rsos.180248)

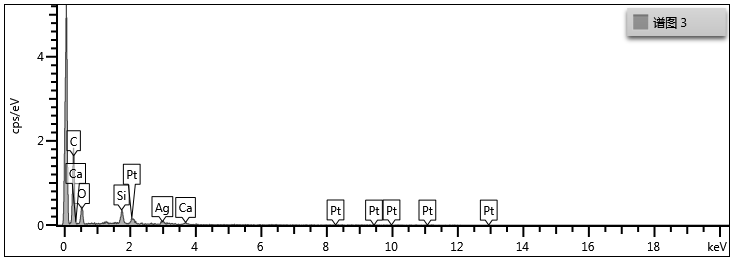


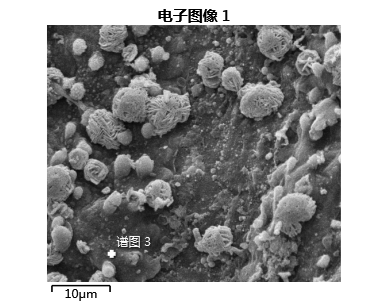

Supplement: Electronic supplementary material [file rsos180248supp2.zip › Original data/SEM-EDS of SRHGC.docx]
